# Exploring the Potentiality of a SERS-Active pH Nano-Biosensor

**DOI:** 10.3389/fchem.2019.00413

**Published:** 2019-06-07

**Authors:** Angela Capocefalo, Daisy Mammucari, Francesco Brasili, Claudia Fasolato, Federico Bordi, Paolo Postorino, Fabio Domenici

**Affiliations:** ^1^Dipartimento di Fisica, Sapienza Università di Roma, Rome, Italy; ^2^Dipartimento di Scienze e Tecnologie Chimiche, Università di Roma Tor Vergata, Rome, Italy; ^3^Dipartimento di Fisica e Geologia, Università di Perugia, Perugia, Italy

**Keywords:** SERS, pH sensor, plasmonics, gold nanoparticles, biosensing, extracellular pH, cancer cells

## Abstract

The merging of the molecular specificity of Raman spectroscopy with the extraordinary optical properties of metallic nanoarchitectures is at the heart of Surface Enhanced Raman Spectroscopy (SERS), which in the last few decades proved its worth as powerful analytical tool with detection limits pushed to the single molecule recognition. Within this frame, SERS-based nanosensors for localized pH measurements have been developed and employed for a wide range of applications. Nevertheless, to improve the performances of such nanosensors, many key issues concerning their assembling, calibration and stability, that could significantly impact on the outcome of the pH measurements, need to be clarified. Here, we report on the detailed characterization of a case study SERS-active pH nanosensor, based on the conjugation of gold nanoparticles with the pH-sensitive molecular probe 4-mercaptobenzoic acid (4MBA). We analyzed and optimized all the aspects of the synthesis procedure and of the operating conditions to preserve the sensor stability and provide the highest responsiveness to pH. Exploiting the dependence of the SERS spectrum on the protonation degree of the carboxylic group at the edge of the 4MBA molecules, we derived a calibration curve for the nanosensor. The extrapolated working point, i.e., the pH value corresponding to the highest sensitivity, falls at pH 5.6, which corresponds to the pKa value of the molecule confined at the nanoparticle surface. A shift of the pKa of 4MBA, observed on the molecules confined at the nanostructured interface respect to the bulk counterpart, unveils the opportunity to assembly a SERS-based pH nanosensor with the ability to select its working point in the sensitivity region of interest, by acting on the nanostructured surface on which the molecular probe is confined. As a proof-of-concept, the nanosensor was successfully employed to measure the extracellular pH of normal and cancer cells, demonstrating the capability to discriminate between them.

## Introduction

In the last decades, the advances in the field of plasmonics enabled to tailor and enhance electromagnetic fields at the subwavelength scale, with a wide range of applications spreading from electronics to biomedicine (Stockman et al., 2018). The opportunity to exploit the nanoscale localization of such intense fields to push the sensitivity of traditional vibrational spectroscopies has raised a great deal of interest around surface enhanced spectroscopies (Camden et al., 2008; Lal et al., 2008). In this context, a relevant position is occupied by Surface Enhanced Raman Spectroscopy (SERS) which, benefiting of the highest signal enhancement [10^6^-10^9^ orders of magnitude (Le Ru et al., 2007)], allows to overcome the intrinsic limit of low intensity of Raman spectroscopy and hence has emerged as one of the most widely used technique in biosensing (Cialla et al., 2014). Recent advances in SERS-based detection methods involve the development of novel chips for immunoassays with enhanced sensitivity and specificity for the study of protein affinity (Chuong et al., 2017) and for the identification of pathogens based on the nucleic acid recognition (Dougan and Faulds, 2012). Beyond the application as ultrasensitive spectroscopy, SERS turned out to be an invaluable technique capable to actively and selectively interact with complex biological systems, including cells and tissues (Fasolato et al., 2016; Cialla-May et al., 2017);(Kneipp, 2017).

In this context, colloidal noble metal nanoparticles play a central role thanks to their high surface-to-volume ratio and to their ease of synthesis and functionalization in different environmental conditions, which make them a versatile platform to develop SERS-active chemical sensors (Yeh et al., 2012). In particular, by employing weak acids as reporter molecule and gold or silver nanoparticles as enhancing scaffold, it has been possible to develop pH sensors capable to convey the SERS potential in providing localized and molecular specific information (Bishnoi et al., 2006; Lawson et al., 2014; Gühlke et al., 2015; Williams et al., 2016). The development of tools for the evaluation of pH at the nanoscale enables to obtain information on small sample volumes, e.g., microfluidic devices or even single cells (Jamieson et al., 2015; Puppulin et al., 2018). In the latter framework, alterations of the local pH of cellular compartments could have dramatic effects on cells and organelles, encouraging the occurrence of diseases. Furthermore, the extracellular pH of cancer cells is expected to be more acidic with respect to that of healthy ones (Webb et al., 2011).

Despite the broad scientific landscape involving the employment of SERS-based pH sensors, many key issues concerning their assembling, calibration and stability, that could significantly affect the precision and accuracy of the pH measurements, need to be clarified.

Here we report on the detailed characterization of a SERS-active pH nanosensor based on the conjugation of gold nanoparticles (AuNPs) with the pH-sensitive molecular probe 4-mercaptobenzoic acid (4MBA), a benzene derivative with a thiol group, which covalently binds to the AuNPs surface, and a carboxylic acid in the opposite position. This molecule is particularly suitable to realize a SERS sensor since the protonation degree of the carboxyl moiety acts as pH indicator (Michota and Bukowska, 2003; Li et al., 2015) that, combined with the strong SERS cross section typical of benzene-derived molecules, results in a spectrum sensible even to weak pH variations (Bishnoi et al., 2006; Williams et al., 2016). Moreover, the orientation with respect to the thiol group provides minimized steric hindrance and maximum exposure outwards of the carboxylic moiety in comparison with other carboxyl-modified benzoic acid derivatives. This allows for a more packed coverage of AuNPs by 4MBA and therefore for a more efficient response to pH variations. With specific reference to the biological application of measuring the cellular pH, the choice of employing of AuNPs as plasmonic scaffold ensures higher chemical stability and biocompatibility with respect to different SERS-active metals such as silver (Wang et al., 2012).

Based on the analyses of the case study of the 4MBA-AuNPs nanosensor, the final purpose of the present work is to develop a systematic and general protocol for the characterization and employment of a nansosensor in different environments. To this aim, all the underlying aspects of the synthesis procedure and of the operating conditions have been analyzed and rationalized to provide the highest sensitivity to pH variations and to preserve the sensor stability. In particular, we focused on the colloidal stability of the system, a crucial parameter for measurements in liquid environment, and on its photostability, identifying a variability range of the measurement parameters to reduce the radiation-induced molecule degradation. All the spectral modifications, directly and indirectly associated to pH variations, have been analyzed and discussed to achieve a close control on all the aspects involved in the measurement.

In the perspective of obtaining a fine tuning of the nanosensor features, particular emphasis has been placed in analyzing the acidic properties of the molecular probe conjugated to the AuNPs in terms of the pKa value. Three calibration curves have been provided in terms of relative intensity of selected pH-dependent SERS bands as a function of the pH and the dynamic range of sensitivity of the nanosensor was identified. The suitability of the 4MBA-AuNPs pH nanosensor for biological applications was demonstrated by measuring the extracellular pH of two cellular models: keratinocyte HaCaT and melanoma SK-Mel5 human skin cells. The possibility to discriminate between the two types of cells highlights a great potential for future diagnosis applications.

## Materials and Methods

### Nanosensor Assembling

AuNPs with nominal diameter of 60 nm stabilized by a citrate capping were purchased from Ted Pella Inc. 4MBA and all the other chemicals involved in sample preparation were purchased from Sigma-Aldrich and used without further purification.

The pH nanosensor employed in this study consists of a core-shell system made up of colloidal AuNPs functionalized by a layer of 4MBA. The molecular functionalization, sketched in Figure S1A, was performed by adding 5 μL of an ethanol solution of 4MBA (0.1 mg/mL) to the water-dispersed colloids stock solution. We employed a large excess of 4MBA molecules to obtain a full coverage of the AuNP (Fasolato et al., 2014), which based on steric hindrance considerations can be esteemed to ~2 × 10^4^ molecules per AuNP. In this way it was possible to ensure the stability of the colloidal dispersion and to maximize the SERS cross section of the nanosensor. In addition, a high coverage of the AuNPs prevents the molecule degradation that might be induced by the interaction of the carboxyl moiety with the gold surface (Michota and Bukowska, 2003), as discussed in detail in section Nanosensor Assembling and Stability. The solution was incubated for 3 h at room temperature and the excess of unbound 4MBA was removed by 24 h dialysis against Milli-Q water, under continuous gentle stirring, using a filter with a cutoff of 50 kDa. A careful characterization was performed after each step and at the end of the functionalization by UV-Visible absorption spectroscopy and Dynamic Light Scattering (DLS).

### UV-Visible Absorption Spectroscopy

The molecular functionalization of AuNPs with 4MBA has been checked by monitoring the absorption peak corresponding to the Localized Surface Plasmon Resonance (LSPR) of the AuNPs by UV-Visible absorption spectroscopy. Being the LSPR very sensitive to the dielectric environment, information on the coverage can be inferred from the observed wavelength shift. Measurements were performed employing a double beam Jasco V-570 Uv/Vis/NIR spectrophotometer, with a resolution of 0.1 nm in the UV-Vis region and 0.5 nm in the NIR region.

### Dynamic Light Scattering

Size and ζ-potential distributions of the water dispersed 4MBA-AuNPs were characterized by Dynamic Light Scattering (DLS). Measurements were performed employing a Malvern NanoZetaSizer apparatus equipped with a 5 mW HeNe laser (Malvern Instruments Ltd, UK) in quasi-backscattering detection, i.e., the scattered light was collected at an angle of 173°. In order to obtain the size distributions, the measured autocorrelation functions were analyzed by the CONTIN algorithm. ζ-potential measurements were performed adopting the Phase Analysis Light Scattering technique of the same Malvern NanoZetaSizer apparatus, by employing a palladium electrode dip cell ZEN 1002 (Malvern, UK). The measured electrophoretic mobility values *μ*_*e*_ were converted into the ζ-potential using the Smoluchowski relation ζ = *μ*_*e*_η*/*ε, where ε is the solvent permittivity and η its viscosity.

### Cell Culture

The cell lines employed in the experiments were obtained from Interlab Cell Line Collection (Istituto Nazionale per la Ricerca sul Cancro, Genoa, Italy) and kindly provided by the Molecular Medicine Department of Sapienza University of Rome. Non-tumorigenic human keratinocyte (HaCaT) and tumorigenic human skin melanoma (SK-Mel5) cell lines were grown, respectively, in Dulbecco's Modified Eagle Medium (DMEM; Euroclone, Life Science Division, GB, Pero, Italy) and Minimum Essential Medium (MEM; Euroclone). All media were supplemented with 1% penicillin/streptomycin, 10% fetal bovine serum and 2 mM L-Glutamine (Euroclone). MEM was also supplemented with 1% non-essential amino acids and 1% Na pyruvate. Before experiments cells were plated onto a glass coverslip deposited in a cell culture Petri dish and then incubated at 37°C under 5% CO_2_.

### Raman Micro-spectroscopy and SERS Measurements

4MBA-AuNPs samples for Raman measurements were prepared as sketched in Figure S1B. A droplet of the solution was deposited on a glass coverslip and dried at room temperature. A representative optical microscopy image of the so obtained substrate, showing self-assembled clusters with micrometric size, is also shown in Figure S1B. SERS measurements were performed in liquid as sketched in Figure S1C, by exposing the dried substrate to solutions with pH adjusted to the desired value. Preliminary stability and reversibility tests have been performed in a broad pH range, spanning between pH 2 and pH 12. SERS measurements of the cellular samples were performed by superimposing a SERS substrate with 4MBA-AuNPs onto a glass coverslip with the cells cultured according to section Cell Culture, as sketched in Figure S9. For each sample at least 10 spectra have been collected.

Raman and SERS measurements were performed employing a Horiba HR-Evolution microspectrometer, equipped with a 15 mW He–Ne laser (632.8 nm wavelength) and a set of neutral power attenuating filters. The spectrometer is coupled with a confocal microscope equipped with a set of objectives at different magnification. In the experiments a 50× –0.50 NA objective (laser spot diameter 1.5 μm) was employed for SERS measurements in liquid and a 100× –0.8 NA objective (laser spot diameter 1 μm) was employed for dried samples. A 600 lines/mm diffraction grating ensures a spectral resolution of 3 cm^−1^. The spectrometer is equipped with an automatic mapping stage with sub-micrometric precision (~0.3 μm).

All the spectra are here presented after a calibration of the Raman shift frequencies obtained by exploiting the emission spectrum of a neon lamp and a polynomial baseline subtraction performed using LabSpec software. To improve the signal-to-noise ratio, the spectra shown in Figure 3 and Figure S5 have been slightly smoothed using a FFT filter procedure (2 points of window). The frequencies of the main Raman and SERS bands were estimated by a fitting procedure performed using Origin 8.1 software.

## Results and Discussion

### Nanosensor Assembling and Stability

In this paragraph we report on all the aspects concerning the fabrication and characterization of the nanosensor with special focus on the colloidal stability, pivotal to refine the performances in liquid environment, and on the chemical alterations that might occur in the molecular probe due to the laser illumination.

The employed nanosensor consists of a plasmonic core made of 60 nm AuNPs conjugated with 4MBA, according to the protocol described in section Nanosensor Assembling. Briefly, as sketched in Figure S1A, the spontaneous S-Au functionalization reaction between the thiol group of the 4MBA and the AuNPs surface was carried out in aqueous solution and thereafter the assembled 4MBA-AuNPs were purified to remove unbound molecules.

The functionalization procedure was monitored by UV-Vis absorption and Raman spectroscopies. Being related to the presence of 4MBA, both the absorption bands in the UV region (Guo et al., 2012) and the redshift of ~4 nm of the LSPR of the AuNPs (LSPR at 533 nm for bare AuNPs), driven by the change in the dielectric environment at the AuNPs surface (Mock et al., 2003), are indicative of the effective conjugation. The absorption spectrum of the 4MBA-AuNPs in comparison with that of the bare AuNPs is reported in Figure S2.

**Figure 1:**
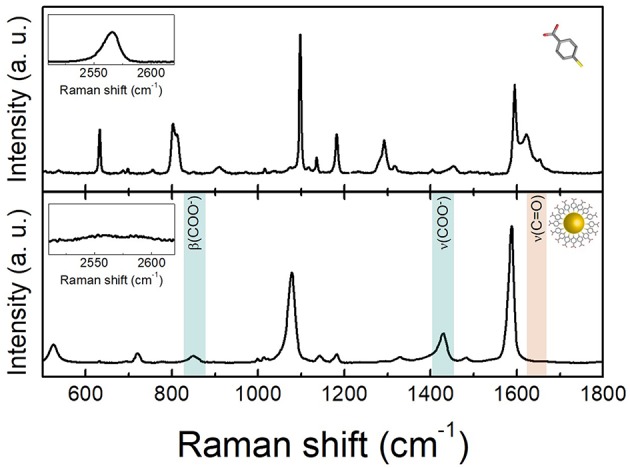
Raman spectrum of bulk 4MBA (top) and SERS spectrum of 4MBA conjugated to AuNPs and dried onto a glass slide (bottom). The peaks of the pH-dependent bands in the SERS spectrum are highlighted: β(COO^−^) and ν(COO^−^) in blue and ν(C = O) in red. The spectral region of the ν(SH) band is reported in the insets. The disappearance of the corresponding peak in the SERS spectrum witnesses a successful functionalization of the AuNPs by S-Au covalent binding.

The Raman spectra of 4MBA-AuNPs and bulk 4MBA, both deposited on a glass slide are shown in Figure 1. The complete assignment of the main Raman and SERS bands is reported in Table 1.

**Table 1:** Peak assignment of the main Raman and SERS bands of the spectra reported in Figure 1 according to Michota and Bukowska (2003), Varnholt et al. (2014), Li et al. (2015) and Williams et al. (2016).

**Peak assignment**	**Raman shift (cm^**−1**^)**	**SERS frequency (cm^**−1**^)**
S-Au	–	228
ν(CS)	–	525
ν_6b_	632	631
β(CO_2_) + ν(CS)	686	–
ν_4b_ + γ(CCC)	–	720
ν_10a_	801	–
ν_17b_ + ω(CO_2_)	812	–
β(COO^−^)	–	849
β(SH)	915	–
ring deformation	–	1,011
ν_12_ – ring breathing	1,098	1,076
ν_19b_ + ν(CS)	1,116	–
ν_9a_	1,135	–
ν(CCOO^−^) + ν(CS)	–	1,143
β(CH)	–	1,183
ν_3_	1,292	–
ν(COO^−^)	–	1,428
ν_8a_ – ring breathing	1,594	1,586
ν(C=O)	1,620	1,710
ν(SH)	2,565	–

*Greek letters indicate the vibrational modes: ν, stretching; β, bending; ω, wagging; γ, out-of-plane vibration. Benzene modes are reported according to the Wilson notation (Wilson, 1934). The rows highlighted in light blue identify the bands whose intensity increases with pH, while the one whose intensity decreases with pH is highlighted in light red. The band corresponding to the S-Au and ν(SH) vibrations, used to assess the functionalization of AuNPs with 4MBA, are highlighted in gray*.

The SERS spectrum of 4MBA-AuNPs is characterized by two intense bands at 1,076 and 1,586 cm^−1^, corresponding to the vibrations of the aromatic ring of the molecule (Michota and Bukowska, 2003), both redshifted with respect to their Raman counterpart. It is known that a relaxation of Raman selection rules occurs when molecules are in proximity to a metal surface, under near-field illumination: this can cause the appearance, in the SERS spectra, of bands that are hindered in the conventional Raman spectra (Moskovits and Suh, 1984; Le Ru et al., 2008, 2011). For analogous reasons, frequency shifts can take place as a result of molecule-metal charge transfer phenomena (Osawa et al., 1994). The successful functionalization by S-Au covalent binding is witnessed by the appearance of the band at 228 cm^−1^ (see Figure S3), corresponding to the gold-sulfur vibration (Varnholt et al., 2014) and by the concomitant vanishing of the band at 2,565 cm^−1^, showed in the insets of Figure 1, corresponding to the thiol S-H stretching vibration (Fasolato et al., 2014). Furthermore, the disappearance of the latter peak certifies that all the molecules concurring to the SERS signal are covalently bound to the AuNPs.

The bands that are sensitive to pH variations are those related to the carboxylic moiety at the edge of the 4MBA molecule, i.e., the bending and stretching vibrations β(COO^−^) and ν(COO^−^) at 849 and 1,428 cm^−1^, and the weak band at 1,710 cm^−1^ corresponding to the ν(C = O) stretching vibration (Li et al., 2015; Williams et al., 2016). The first two bands arise when the carboxylic groups are in the deprotonated form, in basic environment; while the ν(C = O) band appears more intense in acidic condition, when the moiety is protonated. In the SERS spectrum of Figure 1, the bands corresponding to COO^−^ vibrations are prominent, suggesting that most of the carboxylic residues of the 4MBA-AuNPs deposited after the functionalization are in the deprotonated form.

Moving into the issues concerning the actual applicability of the nanosensor, we analyzed the stability of the 4MBA-AuNPs colloidal dispersion by DLS measurements, in terms of size distribution and ζ-potential, and by UV-Visible absorption spectroscopy at varying the pH of the solution. The combined measurements allowed to correlate the aggregation of the system, which occurs upon pH variations due to the related changes in the 4MBA-AuNPs surface charge, with its optical response. The ζ-potential of particles in solution, which is proportional to their average surface charge, is a well-established indicator of the stability of a colloidal dispersion, quantifying the electrostatic repulsion between like-charged particles (Bhattacharje, 2016).

**Figure 2:**
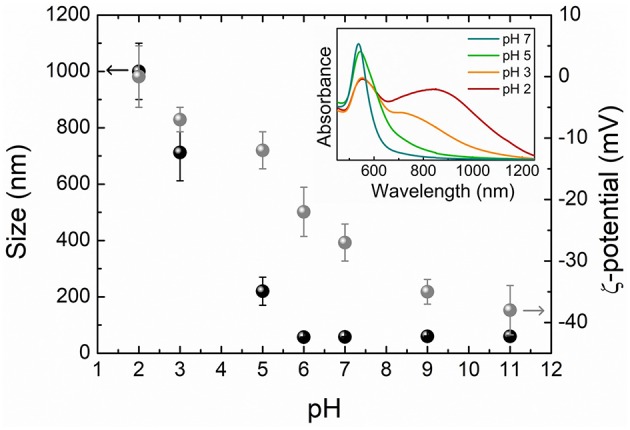
Size (black) and ζ-potential (gray) of 4MBA-AuNPs obtained by DLS measurements as a function of the pH of the solution. The error bars represent the standard deviations of the measured size and ζ-potential distributions. In the inset are reported selected UV-Visible absorption spectra.

Size and ζ-potential results are shown in Figure 2, where a remarkable pH-dependence is clearly observed for both the quantities. At basic pH and down to pH 6, the absolute value of the ζ-potential is high enough (−38 mV < ζ < −22 mV) to prevent the 4MBA-AuNPs aggregation, with size distributions centered on that of single AuNPs. Starting from pH 5, the ζ-potential decreases in modulus and the colloids form aggregates whose dimensions increase with lowering the pH. At pH values lower than 3, 4MBA-AuNPs aggregates reach a micrometric size and could precipitate, as the ζ-potential approaches zero. Notice that the ζ-potential shows the highest slope, corresponding to the maximum sensitivity of the surface charge of the nanosensor with respect to pH variations, in the region between pH 5 and pH 6. Since the 4MBA-AuNPs surface charge basically depends on the protonation degree of the exposed carboxyl moieties, this finding strongly suggests that the working point of the SERS-active nanosensor lies in the same pH range. For the same reason, at pH > 9 the ζ-potential remains essentially unchanged, pointing out that all the carboxylic groups are in the deprotonated form. In these conditions the average number *N* of 4MBA molecules bound to each AuNP is equal to the number of elementary charges on the surface of each 4MBA-AuNPs colloids. This allows to validate the geometrical esteem of the 4MBA coverage (*N* ~2 × 10^4^ molecules per AuNP) using the electrophoretic mobility value (see section Dynamic Light Scattering) measured at pH 11. By employing the Hückel relation it is possible to derive a value for the effective charge *Q*_*eff*_ of the colloids and compare it to that obtained independently from *N* using the model of Aubouy for spherical particles (Aubouy et al., 2003; Bordi et al., 2006). The good accordance between the two values (*Q*_*eff*_ = −90 ± 10*e* and *Q*_*eff*_ = −94*e*, where *e* is the elementary charge) ascertains the reliability of the esteem herein provided.

Selected absorption spectra corresponding to the DLS measurements are reported in the inset of Figure 2. The resonance frequency of the LSPR is strongly influenced by the aggregation of the nanoparticles, leading to the substantial shift and broadening of the plasmons absorption peak due to the coupling among the single particle plasmonic modes of adjacent nanoparticles (Halas et al., 2011). The optical response of the samples changes coherently with the measured aggregation trends. Noteworthy, starting from pH 5, inter-particle plasmonic modes appear at higher wavelength with respect to the LSPR of non-aggregated colloids and the associated absorption band progressively broadens toward the infrared spectral region with the lowering of the pH. These modifications in the shape, amplitude and frequency of the plasmon resonance could critically affect the outcome of the SERS measurement, resulting in possible variations of the spectral enhancement at a given excitation wavelength (Trautmann et al., 2018).

It is therefore evident that the pH of the environment has an impact on the colloidal stability of the nanosensor. The plasmon resonance modifications that occur upon nanoparticles aggregation could compromise the plasmonic efficiency of the enhancing SERS scaffold. Moreover, the precipitation of the sensor upon aggregation at low pH could restrict the range of pH values that allows reliable measurements. Such issues have to be taken into account for properly controlling and interpreting the measurements in liquid environment. The approach adopted in this work to obtain a SERS enhancement independent on the pH of the solution relies in the self-assembly of the 4MBA-AuNPs in micrometric clusters onto glass slides, as shown in Figure S1B. Such aggregates show a stable plasmonic absorption band in the red spectral region (Fasolato et al., 2014; Domenici et al., 2016). The related SERS enhancement is therefore not affected by a different coupling between plasmonic modes upon aggregation, but only by dimensional scale effects of the cluster size which can be overcome by a normalization procedure respect to the intensity of selected pH-independent bands, as discussed below.

Proceeding on this line, a necessary step is to tackle the issue of the nanosensor photostability, and the associated reproducibility of the SERS response. Indeed repeated, extended illumination could cause a degradation of the molecule. Specifically, 4MBA molecules are known to be subject to decarboxylation, i.e., the loss of the carboxylic moiety, as a result of the interaction with plasmonic-active surfaces (Zong et al., 2014; Williams et al., 2016). The occurrence of this phenomenon is recognized in the spectra by the appearance of two new peaks at 996 and 1,019 cm^−1^ (Figure S4), which are attributable to the benzene monosubstituted thiophenol (Michota and Bukowska, 2003). The two bands are mainly related to the ring out-of-plane and in-plane deformations, respectively (see Table S1) (Fontana et al., 2013).

**Figure 3:**
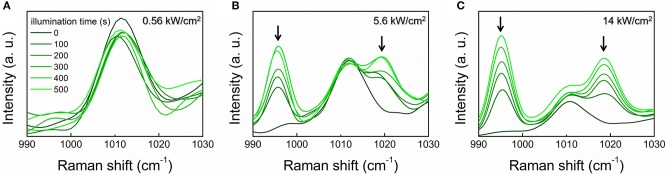
SERS spectra of 4MBA-AuNPs deposited on glass slides over the 990–1,030 cm^−1^ spectral range. The onset of the spectral markers of the irradiation-induced decarboxylation at 996 and 1,019 cm^−1^ (highlighted by the arrows) is well-evident. The spectra were acquired at varying the illumination time with laser intensity of 0.56 kW/cm^2^ **(A)**, 5.6 kW/cm^2^ **(B)** and 14 kW/cm^2^ **(C)**. A FFT filter smoothing was applied to the spectra.

Since the loss of the carboxylic moiety directly affects the nanovector sensitivity to pH variations, we carried out a study with the aim of identifying the experimental conditions to minimize the decarboxylation process. SERS spectra of the 4MBA-AuNPs deposited on glass slides at varying the illumination time and the laser intensity are shown in Figure 3, over the spectral region where are located the spectral markers of decarboxylated 4MBA.

When comparing the reported spectra, a clear dependence of the decarboxylation process on the laser intensity can be recognized. Noticeable, at 0.56 kW/cm^2^ the loss of the carboxylic group does not occur, even upon repeated measurements (Figure 3A). At 5.6 kW/cm^2^, the thiophenol peaks appear already after 100 s illumination (Figure 3B). The observed phenomenon is ascribed to plasmon-derived “hot” electrons extracted from the nanoparticle upon plasmon excitation. These are transferred to the molecule, where they promote the decarboxylation reaction which is also accelerated by the local increase of temperature (Zong et al., 2014).

To deepen the investigation on the decarboxylation phenomenon, we acquired SERS spectra by exposing 4MBA-AuNPs to acidic and basic solutions, as reported in Figure S5. A dependence on the pH of the solution appears evident. In particular, for acidic pH the decarboxylation threshold is shifted to higher laser intensities. This effect is mainly ascribed to the orientation of the 4MBA molecules with respect to the AuNPs surface. At basic pH, indeed, 4MBA preferentially adopts a flat configuration when adsorbed on the surface (Ho and Lee, 2015), promoting the interaction of the deprotonated carboxylic moiety with the gold surface and in turn the plasmon-induced decarboxylation. At acidic pH, on the other hand, the molecules stand upright with respect to the surface, and the carboxylic group is further away from the metal surface.

Having reached a control on the experimental issues concerning the applicability of the nanosensor, in the next section we provide a detailed study of the pH-dependent 4MBA-AuNPs SERS spectra with the final aim of obtaining a calibration of the nanosensor.

### SERS Responsiveness to pH Variations and Calibration of the Nanosensor

The analyses reported in the previous section were aimed to the optimization of the experimental conditions for both the synthesis and the employment of the 4MBA-AuNPs sensor. Here we investigate the SERS response to pH variations of the assembled nanosensor, with the purpose to determine its dynamic range of sensitivity. Proceeding from this, a thorough study concerning the acidic properties of the molecule has been conducted to explore the possibility to obtain a modulation of the working point of the nanosensor.

A preliminary test of the sample responsiveness has been provided by sequentially immersing the nanosensor to solutions with extreme pH values. The results, corresponding to cyclic measurements performed at pH 3 and pH 10 are reported in Figure S6. The features of the spectra show a good responsiveness of the sensor and a high reproducibility for consecutive measurements. In any case, to avoid the presence of salt residues affecting the SERS spectrum (Sun et al., 2015), after each measurement and before the exposure to the next solution, the substrate was rinsed with pure water.

**Figure 4:**
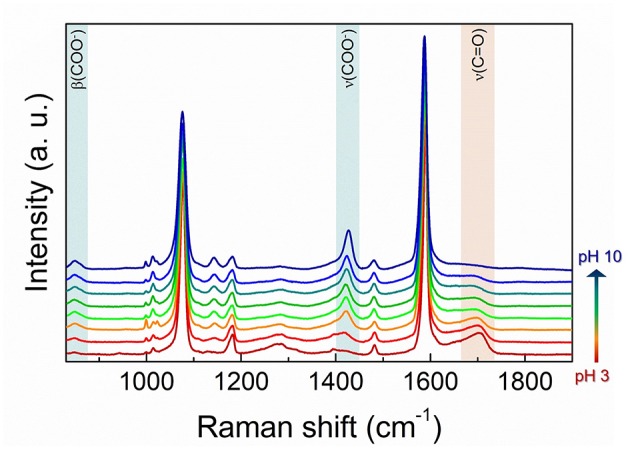
Representative SERS spectra of 4MBA-AuNPs exposed to solutions at different pH. The peaks corresponding to the vibrations of the carboxylic group are highlighted, in the deprotonated (β(COO^−^) and ν(COO^−^), in blue) and protonated case (ν(C = O) in red), respectively.

As starting point for the nanosensor calibration, spectra of 4MBA-AuNPs deposited on a glass slide and exposed to solutions at different pH were acquired. Selected SERS spectra are reported in Figure 4. To better visualize the spectral differences, and to compensate possible effects of the number of molecules concurring to the SERS signal as well as of the SERS enhancement due to the non-homogeneous morphology of the clusters, which affects the signal intensity, all the spectra shown are normalized to the pH-independent integrated intensity of the peak at 1,586 cm^−1^, ascribed to the aromatic ring breathing mode. Only a slight redshift of the peak upon pH increase can be observed, attributable to the coupling with the stretching mode of the carboxyl, more intense in case of deprotonated 4MBA (Liu et al., 2013). Notice that the intensity of the peak at 1,076 cm^−1^, also ascribed to the benzene ring, is automatically normalized.

Clear modifications appear in the spectra upon pH variations from the acidic to the basic pH (from red to blue in the figure). The more pronounced are those occurring in the peaks corresponding to the β(COO^−^) and ν(COO^−^) bending and stretching modes and to the ν(C = O) stretching. As mentioned above, the first two are related to the progressive deprotonation of the 4MBA molecules at high pH that results in a progressive increase in the intensity of the bands related to the COO^−^ group vibrations. On the contrary, with lowering pH, the number of the protonated carboxylic groups COOH increases, resulting in the appearance of the band at 1,710 cm^−1^ which becomes more intense at the lower pH values.

**Figure 5:**
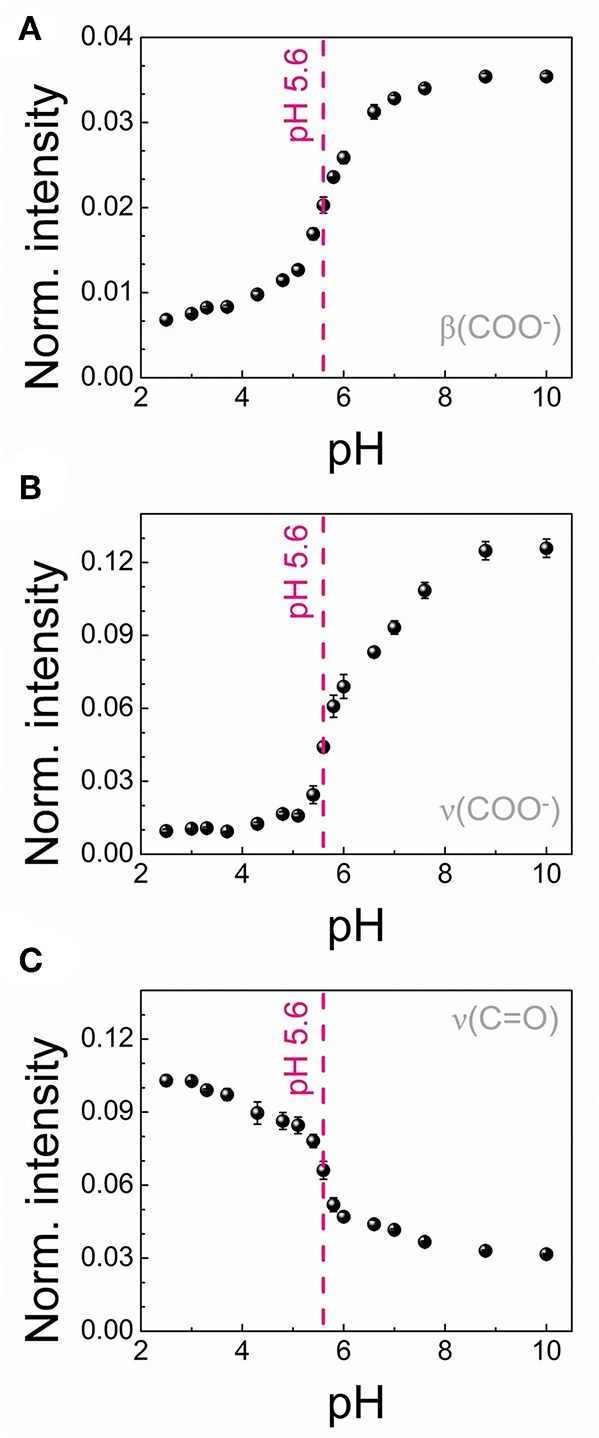
Calibration curves for the 4MBA-AuNPs nanosensor obtained for three different pH-dependent SERS bands: β(COO^−^) bending mode at 849 cm^−1^ **(A)**, ν(COO^−^) stretching mode at 1,428 cm^−1^ **(B)** and ν(C = O) stretching mode at 1,710 cm^−1^ **(C)**. In all the graphs the region of maximum sensitivity respect to pH variations has been indicated.

In order to actually employ the 4MBA-AuNPs as nanosensor for pH measurements, we analyzed the SERS spectra to construct suitable calibration curves. To this end, we calculated the integrated intensities *I* of the above-mentioned pH-dependent peaks in the normalized spectra highlighted in Figure 4. The three calibration curves obtained, reported in Figure 5, show a sigmoidal-like trend, in line with those reported in literature for similar systems (Wang et al., 2012; Lawson et al., 2014; Jamieson et al., 2015). For each curve we determined the working point of the nanosensor, namely the pH value corresponding to the highest sensitivity $\left|\frac{dI}{dpH}\right|$, at pH 5.6, in good agreement with the range identified by ζ-potential measurements. The maximum sensitivity values are 0.017, 0.091, and 0.065 for β(COO^−^), ν(COO^−^), and ν(C = O), respectively. Therefore, amongst all the three curves analyzed, the one which gives the highest sensitivity to pH variations is that derived from the COO^−^ stretching mode (Figure 5B).

With reference to other similar pH nanosensors reported in literature (Jamieson et al., 2015; Wei et al., 2016; Williams et al., 2016; Puppulin et al., 2018), showing a region of sensitivity in the pH range 7–8, the sharp working range identified for our nanoscale pH sensor turns out to be more suitable for monitoring pH-dependent biological processes on single cells. In fact, variations of cellular pH are generally identified at pH values between 5 and 7 (Casey et al., 2010). Going to analyze the chemical aspects in detail, the working range extrapolated from the SERS calibration curves corresponds to the dissociation constant, namely the pKa, of the indicator employed as probe (Wencel et al., 2014). Moreover, the confinement of molecules at nanostructured interfaces could affect their acidic properties, depending on the surface curvature (Leopold et al., 2002; Wang et al., 2011; Koivisto et al., 2016). To deepen this aspect, we performed a standard acid-base titration curve on the bulk 4MBA molecule, reported in Figure S8, obtaining a pKa of 4.2 pH units for the carboxylic acid. The SERS-based calibration curve and the ζ-potential measurements, instead, highlight a weakening of the acidic properties of the 4MBA when bound to the plasmonic nanostructure. Since the deactivation of the thiol moiety upon conjugation with the AuNPs does not affect the acidic properties of the system, as discussed in section Acid-Base Titration of the Bulk 4MBA of Supplementary Material, the observed pKa shift is attributable only to the confinement of 4MBA molecules on the nanostructured surface. Interestingly, this latter aspect points out the possibility of modulating the pKa value of the nanosensor by acting on the properties of the nanostructured surface on which the molecular probe is confined.

The overall modifications that occur in the SERS spectra depending on the pH, not only supply the information that can be used to determine the nanosensor performances but provide unique molecular and stereochemical information which are related as well to the pH variation. With reference to Figure 4, it is possible to notice the appearance of a further peak moving to basic pH, centered at 1,143 cm^−1^, mainly assigned to the ν(CCOO^−^) stretching mode (Liu et al., 2013). Moreover, a modification of the shape of the ν(COO^−^) peak at ~1,400 cm^−1^ is observed with increasing pH. This is due to the increasing weight of a second component of the band, centered at ~1,380 cm^−1^, related to the vibrations at lower energy of the COO^−^ groups that are interacting with the metallic surface. The parallel orientation of the 4MBA molecules with respect to the AuNPs surface, resulting in the COO^−^ group being closer to the gold interface, is in fact favored at higher pH (Michota and Bukowska, 2003) due to the negative charge of the carboxylate. This effect is reflected also in the appearance of a band at 720 cm^−1^, assigned to the γ(CCC) out-of-plane ring vibrations, also index of flat-oriented 4MBA molecules (Ho and Lee, 2015) (see Figure S7).

The great deal of information that can be inferred over the whole fingerprint region reduces considerably the presence of artifacts that might occur with potentiometric methods or paper-based sensors (Khan et al., 2017).

### Nanosensor Application: *In vitro* pH Detection of Living Cells

The calibration curves obtained for 4MBA-AuNPs point out that the dynamic working range of the nanosensor lies in the pH range 5–7, suggesting a high reliability for pH measurements at the single cell level. Therefore, as a proof-of-concept to demonstrate the actual applicability of our SERS-active pH nanosensor we measured the extracellular pH (pH_e_) of two clinically relevant human skin cell lines: normal HaCaT and cancer SK-Mel5. The pH_e_, being involved in cell progression, differentiation and proliferation (Sun et al., 2015), is an important parameter that allows to discriminate unhealthy cells from healthy ones (Neri and Supuran, 2011). Indeed, cancer cells lead to an acidification of the extracellular milieu due to their enhanced glucose metabolism (Neri and Supuran, 2011; Damaghi et al., 2013).

**Figure 6:**
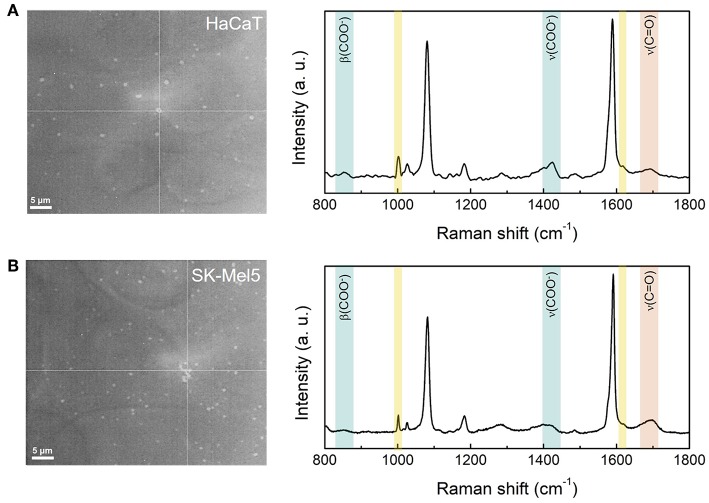
Optical microscope images and SERS spectra of HaCaT **(A)** and SK-Mel5 **(B)** cells exposed to the nanosensor. The bright spots are the 4MBA-AuNPs clusters assembled onto the glass coverslip superimposed to the cells. The grids in the images identify the spatial point corresponding to the SERS spectra. The pH-dependent SERS peaks are highlighted in blue (β(COO-) and ν(COO-)) and in red (ν(C = O)), while the spectral markers of the cell (Phenylalanine at 1,000 and 1,610 cm^−1^) are highlighted in yellow.

According to section Cell Culture, the cells were cultured on a glass coverslip and, for the SERS measurements, the cells were put in contact with the nanosensor by superimposing a second glass coverslip with the 4MBA-AuNPs previously assembled, as sketched in Figure S9. Figure 6 shows two optical microscopy images of the HaCaT and SK-Mel5 cells where the 4MBA-AuNPs clusters are clearly visible. Corresponding SERS spectra collected on the cells are also shown in Figure 6, right side.

The SERS spectra of the 4MBA-AuNPs in contact with the cells do not show spectral distortions and are in excellent agreement with the 4MBA-AuNPs spectra reported in the previous sections. Noteworthy, the peaks corresponding to the ring vibrations of Phenylalanine, at 1,000 and 1,610 cm^−1^ (see Table S2 for the complete peak assignment), can be clearly identified due to the huge Raman and SERS cross section of this amino acid (Cialla et al., 2014). The two peaks can be hence considered as cellular spectral markers (see Figure S10), certifying that the pH nanosensor is effectively in contact with the cells. For each sample, the integrated intensities of the pH-dependent SERS bands in the normalized spectra, according to section SERS Responsiveness to pH Variations and Calibration of the Nanosensor, have been determined to extrapolate the pH_e_ from the calibration curves. The obtained results for each of the three bands are reported in Table 2, together with the standard deviations.

**Table 2:** Estimated pH_e_ values for the normal HaCaT and cancer SK-Mel5 cell lines, extrapolated from the SERS spectra.

	**β(COO^**−**^)**	**ν(COO^**−**^)**	**ν(C=O)**
HaCaT	7.0 ± 0.4	7.4 ± 0.7	6.5 ± 0.9
SK-Mel5	5.6 ± 0.2	6.3 ± 0.6	5.6 ± 0.2

The distributions of the obtained pH values point out that our 4MBA-AuNPs nanosensor yields a good esteem of the pH_e_ (Casey et al., 2010) and, even better, it is suitable to discriminate between normal and cancer cells. In fact, for each of the pH-dependent SERS bands, the measured pH_e_ of the cancer SK-Mel5 cells results to be more acidic respect to that of the normal HaCaT cells. Analyzing in detail the obtained pH_e_ values, the ν(COO^−^) peak systematically yields a slightly higher pH value if compared with the β(COO^−^) and the ν(C = O) peaks. This can be ascribed to the superimposition with the band corresponding to the CH_2_ deformation of the cell membrane lipids. Since the SERS probe is located in proximity of the cell membrane, the spectral contribution of this band results convoluted to that of the ν(COO^−^) band, leading to a slight overestimation of the integrated intensity. Concerning the spectral region in which are located the other two pH-dependent SERS bands, at 853 cm^−1^ it is only present a narrow and not intense peak, ascribed to the Tyrosine ring breathing and, at higher frequencies, the amide I band at 1,660 cm^−1^ is usually suppressed in SERS spectra (Kurouski et al., 2013). These considerations implies that the β(COO^−^) and the ν(C = O) peaks are not affected by the spectral contribution of the cells and allow to obtain a measurement of the pH_e_ from the average of the values reported in Table 2, which is equal to 6.8 ± 0.5 for the HaCaT and to 5.6 ± 0.1 for the SK-Mel5 cells. More in general, the derivation of three different calibration curves for the pH evaluation based on the SERS measurement allows to choose the most appropriate, addressing issues associated with the superimposition of spectral band of the sample and of the probe.

Based on these evidences, we can conclude that our 4MBA-AuNPs pH nanosensor is effectively suitable to perform localized pH measurements on single cells and, even more appealing, it has the potential to distinguish between normal and cancer cells.

## Conclusions

In this work we have presented a detailed study of a SERS-active pH nanosensor made of AuNPs conjugated with the pH-sensitive molecular probe 4MBA, aimed to improve the reliability and accuracy of pH measurements at the nanoscale, providing a thorough characterization of the 4MBA-AuNPs performances in different measurement conditions.

Special focus has been paid to the reproducibility of the SERS response by analyzing how the colloidal properties of the nanosensor could affect the plasmonic efficiency of the SERS enhancing scaffold. Moreover, the experimental condition to minimize the laser-induced degradation of the molecular probe have been identified as well as at varying the pH of the environment.

The calibration of the nanosensor was performed in terms of the intensities of selected pH-dependent SERS bands, identifying the dynamic range of sensitivity centered in the pH range 5–7. The detailed analysis of the whole SERS fingerprint allowed to identify highly correlated spectroscopic markers which provide robust reliability to the measurements.

The comprehensive study of the acidic properties of the molecular probe allowed to reveal a shift of the molecule pKa at the interface of the plasmonic nanostructure. This last finding points out the opportunity to assemble a pH nanosensor with the ability to select its working point in the sensitivity region of interest, depending on the properties (e.g., curvature, material, additional ligand, position of the carboxyl substituent, etc.) of the nanostructured surface on which the molecular probe is confined.

The experimental efforts conducted in this work allowed to successfully employ the 4MBA-AuNPs nanosensor to detect the extracellular pH of normal HaCaT and cancer SK-Mel5 cells, proving the capability of the nanosensor to discriminate between the two cell types. The overall results presented in this work pave the way for realizing efficient SERS-active microplate substrates suitable for biomedical applications, offering the possibility to perform localized pH measurements at sub-cellular level.

## Data Availability

All datasets generated for this study are included in the manuscript and/or the Supplementary Files.

## Author Contributions

FD designed the research and work coordination. PP and FeB provided the equipment. AC and DM performed the research. AC, DM, FrB, CF, PP, and FD analyzed data. AC, FrB, and FD wrote the manuscript. All authors reviewed the manuscript.

### Conflict of Interest Statement

The authors declare that the research was conducted in the absence of any commercial or financial relationships that could be construed as a potential conflict of interest.
