# Supplementary material for: Exploring the Potentiality of a SERS-Active pH Nano-Biosensor
Source: Front Chem. 2019 Jun 7;7:413. doi: 10.3389/fchem.2019.00413 (PMC6568054; doi:10.3389/fchem.2019.00413)
Supplement: Supplementary file 1 [file Data_Sheet_1.PDF]

Supporting Information

Section S1
Nanosensor assembling and characterization

The experimental protocol employed to synthesize the 4MBA-AuNPs nanosensor, to assemble it on a glass slide and to perform SERS measurements is sketched in Figure S1. The UV-Visible absorption spectra before and after the functionalization of AuNPs with 4MBA are reported in Figure S2, highlighting the redshift of the AuNPs LSPR associated to the variation of the refractive index at the metal interface upon covering with the 4MBA layer. The formation of the S-Au bond is assessed by the peak at 228 cm^{-1} in the SERS spectrum of 4MBA-AuNPs reported in Figure S3.

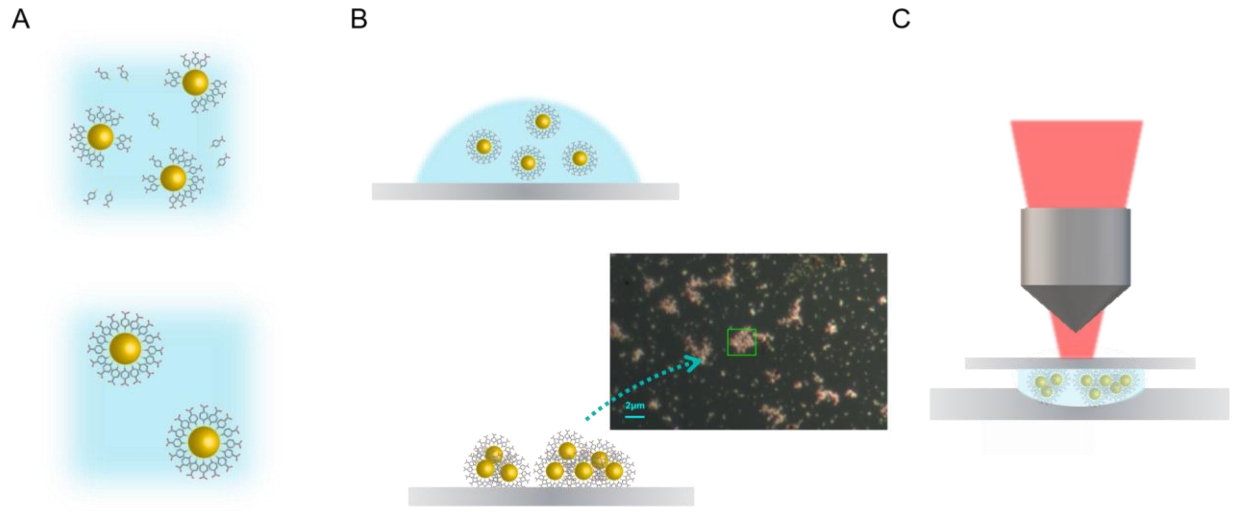

Figure S1: Scheme of the experimental protocol employed to synthesize the 4MBA-AuNPs nanosensor and to perform the SERS measurements. The functionalization reaction between 60 nm AuNPs and 4MBA molecules was conducted in water for 3 h and the unbound molecules were removed by 24h dialysis against MilliQ water (A). A droplet of the 4MBA-AuNPs solution was deposited on a glass coverslip and dried at room temperature. A representative optical microscopy image (scale bar 2 μm) of the self-assembled clusters with micrometric size, is reported. (B). SERS measurements were performed by exposing the glass substrate with the self-assembled 4MBA-AuNPs to solutions at different pH in a concave glass slide (C).

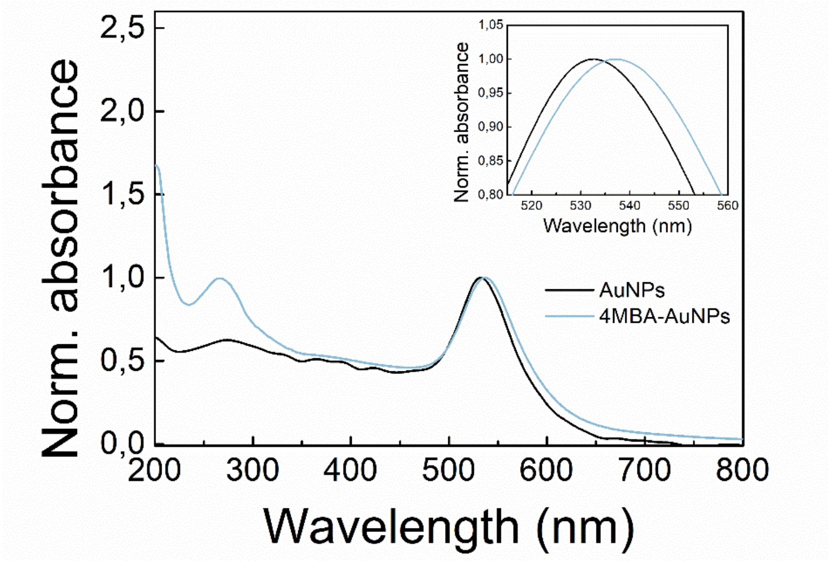

Figure S2: UV-Visible absorption spectra of AuNPs before (black curve) and after (light blue curve) the functionalization with 4MBA. In the inset is reported a zoom of the peak corresponding to the LSPR of the AuNPs, normalized to the maximum absorbance value. The measured redshift of ~4 nm from 533 to 537 testifies a successful functionalization.

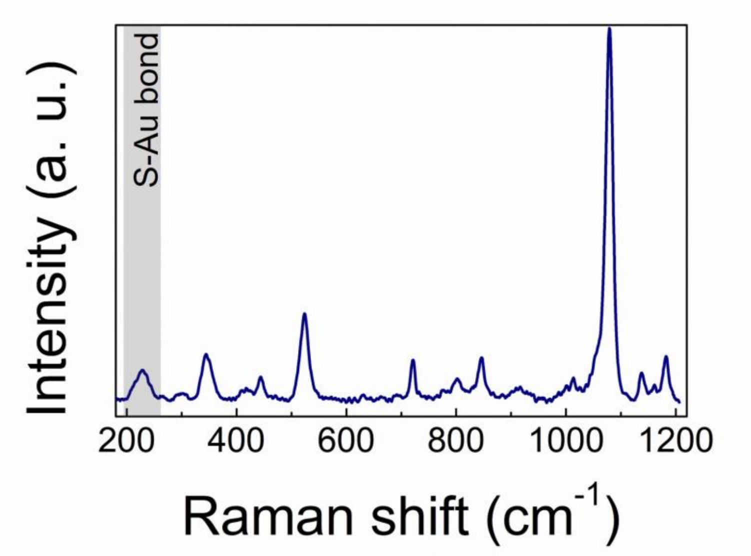

Figure S3: SERS spectrum of 4MBA-AuNPs dried onto a glass slide. The peak at 228 cm^{-1} (highlighted in grey), corresponding to the S-Au vibration assesses the successful functionalization of the AuNPs.

Section S2
Plasmon-induced decarboxylation of 4MBA molecules

In this section are reported the analyses aimed at assessing the stability of the nanosensor with respect to the plasmon-induced decarboxylation of 4MBA molecules. A representative spectrum of the sensor underwent degradation is reported in Figure S4, where the bands unveiling the presence of benzene monosubstituted thiophenol are highlighted. The assignment of the SERS bands of thiophenol according to ref. [Fontana et al., 2013] is reported in Table S1. In Figure S5 is reported the SERS study of the decarboxylation process as a function of the illumination time and of the laser power performed in basic and in acidic environment.

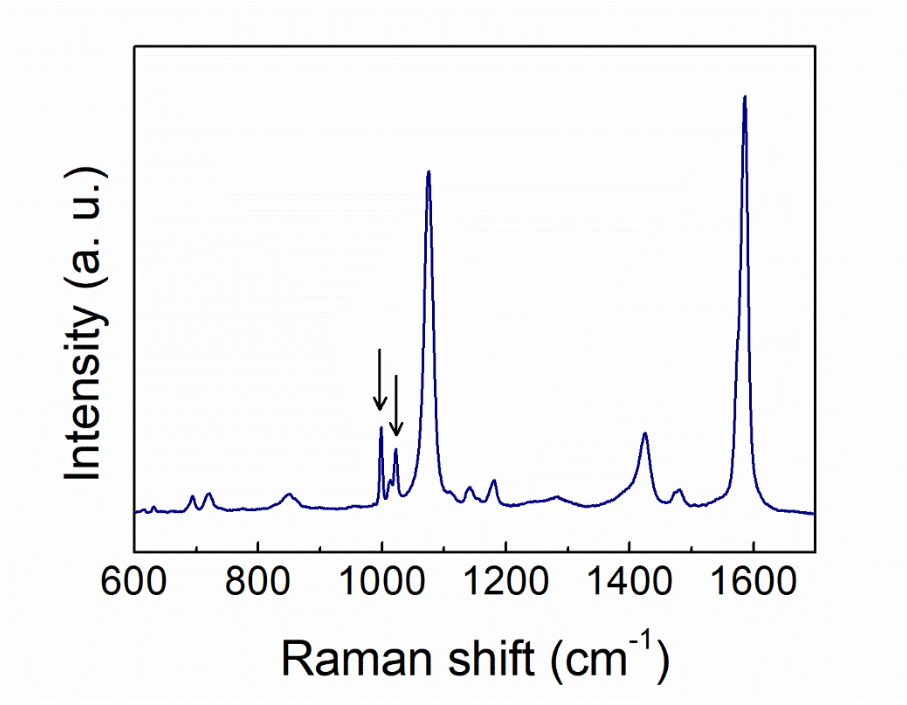

Figure S4: SERS spectrum of 4MBA-AuNPs acquired using the 50× objective with illumination time of 300 s and laser intensity of 14 kW/cm^2; the peaks ascribed to the thiophenol are particularly evident.

Table S1: Peak assignment of the SERS spectra of thiophenol according to [Fontana et al., 2013]. Greek letters indicate the vibrational modes: ν, stretching; β, bending. The rows highlighted identify the SERS bands used as spectral markers of the decarboxylation.

**Peak assignment**	**SERS frequency (cm^{-1})**
ν(CS)	420
ring in-plane deformation + ν(CS)	690
ring out-of-plane deformation + β(CH)	996
ring in-plane deformation + ν_{symm}(CC)	1019
$\nu_{anti\text{-}symm}$(CC) + ν(CS)	1071
ν_{symm}(CC)	1571

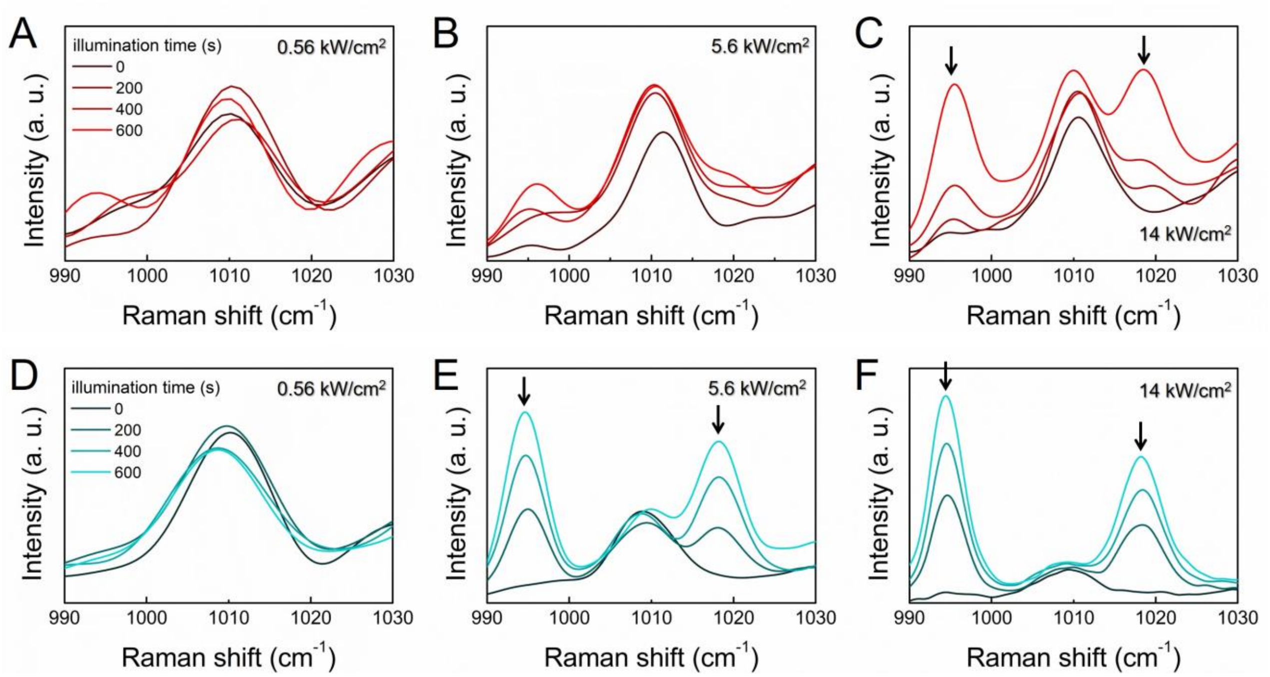

Figure S5: SERS spectra of 4MBA-AuNPs deposited on a glass slide and exposed to solution at acidic (pH 2, top) and basic (pH 10, bottom) pH. The onset of the spectral markers of the irradiation-induced decarboxylation at 996 cm^{-1} and 1019 cm^{-1} are highlighted by the arrows. The spectra are acquired at varying the illumination time with laser intensity of 0.56 kW/cm^2 (A, D), 5.6 kW/cm^2 (B, E) and 14 kW/cm^2 (C, F). A FFT filter smoothing was applied on the spectra.

Section S3
SERS response to pH variations

The SERS response of the 4MBA-AuNPs nanosensor upon alternate exposure to solution at extreme pH values (pH 3 and pH 10) is reported in Figure S6. In Figure S7 is highlighted the intensity increase with the pH of the γ(CCC) peak.

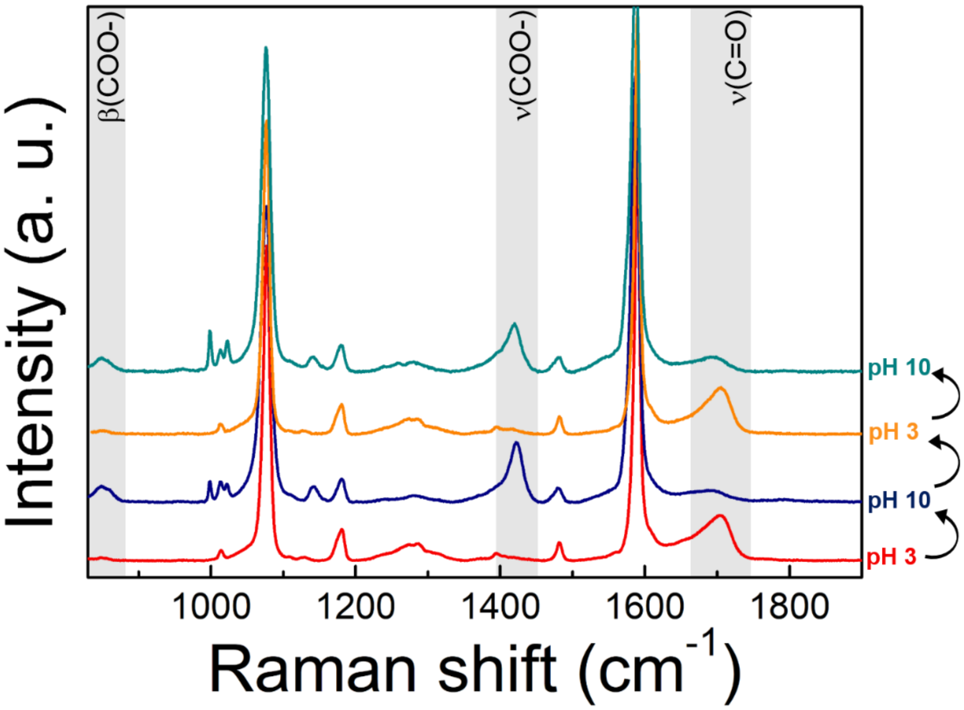

Figure S6: SERS spectra of 4MBA-AuNPs acquired on samples alternately exposed to solutions at pH 3 and pH 10. The pH-dependent peaks have been highlighted. It should be noticed that the occurrence of the decarboxylation (appearance of the peaks around 1000 cm^{-1}), promoted at basic pH, is restricted to the region corresponding to the laser spot.

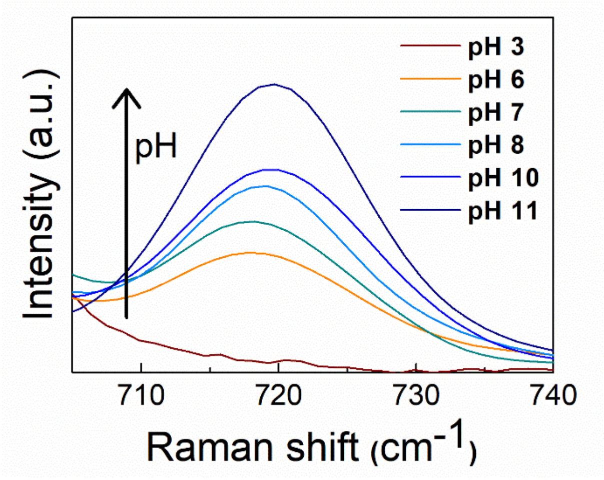

Figure S7: Zoom of the SERS spectra of 4MBA-AuNPs acquired for different pH values in the spectral region corresponding to the γ(CCC) peak.

Section S4
Acid-base titration of the bulk 4MBA

A standard acid-base titration curve was performed on water dispersed 4MBA molecules by employing the following protocol. A solution of 4MBA dissolved in ethanol was diluted in MilliQ water. Acid-base titration was performed by adding 0.05 M NaOH in steps of 5 μL to the solution of 4MBA. After each step, the pH of the solution was measured using a pH meter (Crison) equipped with a micro-pH electrode (XS Instruments, Giorgio Bormac). Before the measurements, the pH-meter was calibrated using pH 4 and pH 7 H_2O-based buffer solutions. The titration curve of 4MBA, reported in Figure S8, was obtained by plotting the measured pH of the solution as a function of the volume of the adding titrant.

In general, the vertical inflection point of a titration curve is called equivalence point and identifies the pH value at which all the acid is converted in its conjugate base. The pKa value of the acid is obtained from the half-equivalence point, located where the volume of the titration agent is halved respect to that of the equivalence point. In fact, at this point the concentration of the acid is the same of that of the conjugate base, thus following the Henderson-Hasselbalch equation the pH of the solution is equal to the pKa of the acid [Koivisto et al., 2016].

The acid-base titration curve of 4MBA yields two equivalence points, at pH 8.4 and at pH 5.2, corresponding to the thiol and to the carboxyl moiety, respectively. The derived pKa values are thus 5.4 and 4.2, consistent with those reported in literature for the 4MBA [Koivisto et al., 2016]. Moreover, the reported value of the pKa of the benzoic acid is also 4.2 [Hollingsworth et al., 2002], thus ensuring that the presence of the sulfhydryl moiety does not affect the acid properties of the carboxyl.

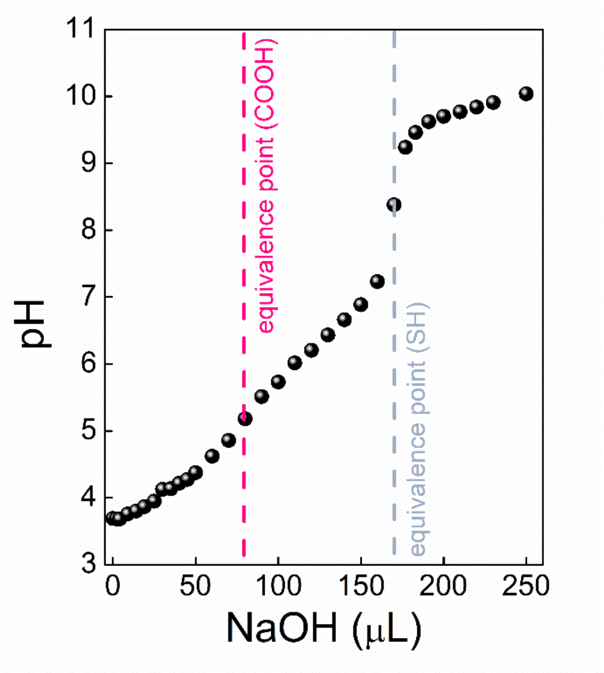

Figure S8: Acid-base titration curve for free 4MBA molecule performed employing NaOH as titration agent.

Section S5
Raman measurements on HaCaT and SK-Mel5 cells

A sketch of the experimental setup employed for the SERS measurement on living cells is reported in Figure S9. Control Raman spectra of HaCaT and SK-Mel5 cells are reported in Figure S10. The complete band assignment is reported in Table S2.

Figure S9: Sketch of the experimental setup employed for the SERS measurements: a glass coverslip with previously assembled 4MBA-AuNPs was superimposed on a second glass coverslip where the cells were cultured.

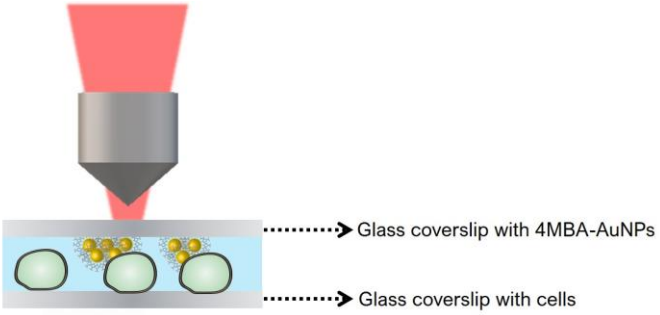

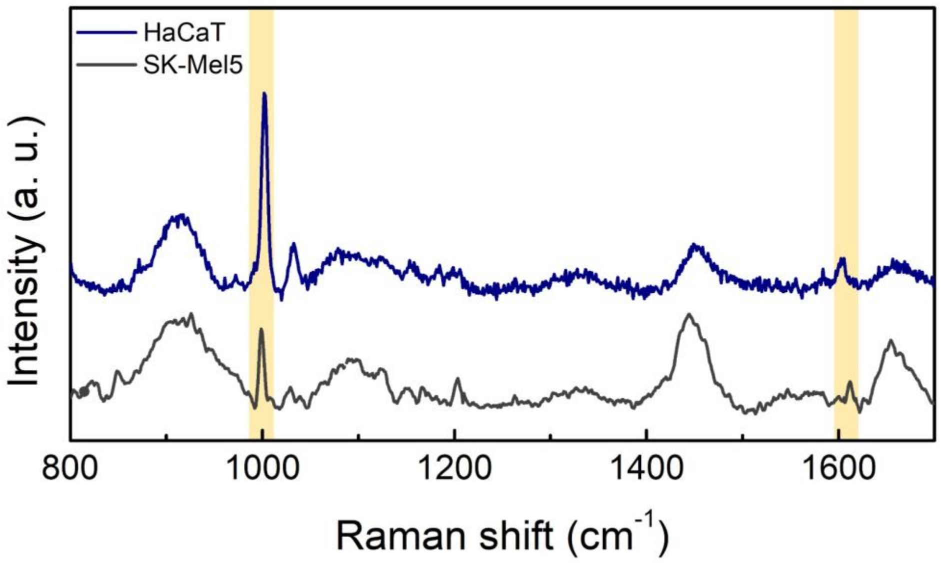

Figure S10: Raman spectra of HaCaT (blue) and SK-Mel5 (grey) cell lines. The peaks corresponding to the Phenylalanine have been highlighted in yellow.

Table S2: Peak assignment of the Raman spectra of cells according to [Notingher et al., 2002; Movasaghi et al., 2007]. The rows highlighted in yellow identify the spectral markers of the cells recognized in the SERS spectra of Figure 6 of the main text.

Peak assignment	Raman shift (cm^{-1})
Tyrosine	640
Tyrosine	853
Phenylalanine	1000
Phenylalanine	1027
PO_2^- symmetric stretching	1093
Adenine + Amide III	1252
Adenine + CH_2 deformation	1313
Lipid CH_2 deformation	1445
Phenylalanine	1610
Amide I	1660

References

[Fontana et al., 2013] J. Fontana, J. Livenere, F. J. Bezares, J. D. Caldwell, R. Rendell and B. R. Ratna (2013) Large surface-enhanced Raman scattering from self-assembled gold nanosphere monolayers; Applied Physics Letters 102, 201606

[Koivisto et al., 2016] J. Koivisto, X. Chen, S. Donnini, T. Lahtinen, H. Häkkinen, G. Groenhof and Mika Pettersson (2016). Acid-Base Properties and Surface Charge Distribution of the Water-Soluble Au102(pMBA)44 Nanocluster; Journal of Physical Chemistry C 120, 10041-10050.

[Hollingsworth et al., 2002] Hollingsworth, C. A., Seybold, P. G., & Hadad, C. M. (2002). Substituent effects on the electronic structure and pKa of benzoic acid. International journal of quantum chemistry, 90(4-5), 1396-1403.

[Notingher et al., 2002] Notingher, I., Verrier, S., Romanska, H., Bishop, A. E., Polak, J. M., & Hench, L. L. (2002). In situ characterisation of living cells by Raman spectroscopy. Journal of Spectroscopy, 16(2), 43-51.

[Movasaghi et al., 2007] Movasaghi, Z., Rehman, S., & Rehman, I. U. (2007). Raman spectroscopy of biological tissues. Applied Spectroscopy Reviews, 42(5), 493-541.
